# *Pasteurella multocida* Toxin Manipulates T Cell Differentiation

**DOI:** 10.3389/fmicb.2015.01273

**Published:** 2015-11-19

**Authors:** Dagmar Hildebrand, Klaus Heeg, Katharina F. Kubatzky

**Affiliations:** Zentrum für Infektiologie, Medizinische Mikrobiologie und Hygiene, Universitätsklinikum HeidelbergHeidelberg, Germany

**Keywords:** Th17 cells, regulatory T cell, STAT proteins, *Pasteurella multocida* toxin, T cell proliferation, T helper cell differentiation, Foxp3, RORγt

## Abstract

*Pasteurella multocida* causes various diseases in a broad range of wild and domestic animals. Toxigenic strains of the serotypes A and D produce an AB protein toxin named *Pasteurella multocida* toxin (PMT). PMT constitutively activates the heterotrimeric G protein subunits Gα_q_, Gα_13_, and Gα_i_ through deamidation of a glutamine residue, which results in cytoskeletal rearrangements as well as increased proliferation and survival of the host cell. In human monocytes, PMT alters the lipopolysaccharide (LPS)-induced activation toward a phenotype that suppresses T cell activation. Here we describe that the toxin also modulates CD4-positive T helper (Th) cells directly. PMT amplifies the expansion of Th cells through enhanced cell cycle progression and suppression of apoptosis and manipulates the differentiation of Th subclasses through activation of Signal Transducers and Activators of Transcription (STAT) family members and induction of subtype-specific master transcription factors. A large population of toxin-treated T cells is double-positive for Foxp3 and RORγt, the transcription factors expressed by Treg and Th17 cells, respectively. This suggests that these cells could have the potential to turn into Th17 cells or suppressive Treg cells. However, in terms of function, the PMT-differentiated cells behave as inflammatory Th17 cells that produce IL-17 and trigger T cell proliferation.

## Introduction

An effective T cell-driven immune response against microbial pathogens depends on the T cell receptor (TCR)-mediated expansion of antigen-specific T cells as well as the differentiation of specialized T cell subsets. The nature of the invading pathogen determines the resulting CD4-positive Th subtype that is generated. Microbial components are recognized by distinct pattern recognition receptors (PRRs) on innate immune cells. As a consequence, professional antigen-presenting cells (APCs) perform phagocytosis and present foreign antigens on major histocompatibility complexes (MHC) to T cells. Binding of presented antigens to the TCR transmits the activation signal to intracellular molecules, which trigger cellular proliferation. In addition, APCs can express T cell-activating surface molecules that bind the co-receptor CD28. This ligand-receptor binding is required for the full activation and expansion of T cells. Depending on the activated PRR, APCs produce a specific set of cytokines that defines the direction of Th differentiation. The released cytokines bind to their responding receptors on the Th cell and induce signaling cascades that are transmitted through Signal Transducers and Activators of Transcription (STAT) proteins. Depending on the cytokine STAT-3, STAT-4, STAT-5, or STAT-6 then induce the expression of Th subtype-specific master transcription factors. Together with the STAT proteins, they finally determine the differentiation of effector cells by triggering gene expression of lineage-characteristic cytokines and surface molecules (O’Shea et al., 2011). In this way, pathogen-specific Th effector cells develop to help provide an immune response tailored to recognize and destroy the microorganism. Initially, only two resulting Th subtypes, Th1 and Th2, were known. While Th1 cells that release IFN-γ and TNF-α, stimulate innate and T cell-induced immunity to recognize intracellular bacteria, Th2 cells boost the response against extracellular pathogens in the humoral and mucosal immunity. Today, a much higher variety of effector Th cells such as Th3, Th9, TR1, T follicular helper cells, Th17 and the suppressive regulatory T cells (Tregs) are known (Zhu and Paul, 2010). They can be seen as separate types or as a specific state of a certain main lineage. The plasticity of T cell differentiation is remarkable and allows a quick adaption to the invading microbe. Furthermore, this plasticity allows the control of the sensitive balance of defense activation and suppression, which is a prerequisite for a successful and moderate immune response. Lately it has become clear that the interplay between Th17 cells and Tregs is particularly important to maintain homeostasis (Astry et al., 2015; Chen et al., 2015; Talaat et al., 2015) as these two T cell subtypes have opposite functions in the regulation of the immune system. Th17 cells are named after the IL-17 family of cytokines and activate a broad range of immune cells (Park et al., 2005), hence Th17 cells are considered potent inflammatory cells with a role in autoimmune disorders (reviewed in (Korn et al., 2009). In contrast, induced Tregs (iTregs) mediate immune suppression and protect from an overactive immune response (Shevach and Thornton, 2014), whereas natural Tregs (nTregs) develop from autoreactive thymocytes in the medulla of the thymus and sustain tolerance to self-antigens (Bettini and Vignali, 2010). The precise division between nTreg and iTreg-mediated modes of suppression however, is still under investigation (Curotto de Lafaille and Lafaille, 2009). Although Th17 cells and Tregs have opposite functions, the differentiation of both lineages is closely connected. Th17 cell development is mediated by TGF-β and IL-6, the activation of STAT-3 and the following induction of RORγt (Ivanov et al., 2006; Tanaka et al., 2014). Induced Tregs can be differentiated from peripheral CD4^+^CD25^-^ T cells through activation of the transcription factors STAT-5 and Foxp3 in the presence of TGF-β and IL-2 (Burchill et al., 2007; Schmitt and Williams, 2013). Thus Th17 differentiation, as well as Treg formation, are dependent on TGF-β. In addition, the transcription factors Foxp3 and RORγt can influence each other and generate intermediate T cell subtypes such as IL-17-releasing Foxp3-positive cells (Voo et al., 2009; Kryczek et al., 2011). Finally, Th17 cells can turn into iTregs and nTregs can be converted into Th17 cells, respectively, under inflammatory conditions (Kong et al., 2012). However, this plasticity of CD4-positive T cells also has disadvantages for the host as it represents a welcome target for pathogens to modulate the immune response. T cell differentiation can either be indirectly modulated through the manipulation of APCs or directly through induction of signaling cascades in the lymphocyte (Miethke et al., 1994; Smith et al., 2004; Ivars, 2007; Sakai et al., 2010; Rodriguez-Garcia et al., 2011). *Pasteurella multocida* toxin (PMT) from *P. multocida* manipulates the host’s signaling cascades through constitutive activation of the alpha subunits of the heterotrimeric G proteins Gq, G13, and Gi through deamidation of glutamine residue (Orth and Aktories, 2010). Downstream of the activated G protein, signaling events such as calcium mobilization, phospholipase C activation, activation of the small GTPase RhoA or mitogen-activated protein (MAP) kinase pathways have been reported (Wilson and Ho, 2011). Furthermore, we could show that PMT mediates long-term activation of the Janus kinase (JAK)-STAT pathway through constitutive activation of Gq. This results in constitutive STAT-3 activation and aberrant expression of its target gene, the kinase Pim-1 which in turn phosphorylates and thus inhibits the negative feedback regulator supressor of cytokine signaling (SOCS)-1 (Orth et al., 2007; Hildebrand et al., 2010). Eventually this causes an enhanced expression of JAK2 and an increase in STAT-3 activity. As a consequence of PMT-induced signaling, the toxin stimulates proliferation of a variety of cells types such as fibroblasts, bladder epithelial cells or osteoclasts, and protects cells efficiently from apoptosis (Preuss et al., 2010; Wilson and Ho, 2012). Recently we have shown that PMT alters the lipopolysaccharide (LPS)-induced activation of human monocytes toward a phenotype which shows decreased T cell activation (Hildebrand et al., 2012). The study presented here aimed to answer the question whether PMT can also directly modulate CD4-positive T cells responses. Our experiments on human blood-derived Th cells demonstrate that the toxin amplifies the CD3/CD28-induced expansion of T cells through enhanced cell cycle progression and survival. Moreover, PMT modulates the differentiation of Th cells into specific subtypes. The toxin mediates activation of STAT family members and the expression of the subtype determining transcription factors RORγt and Foxp3. Our experiments show that the PMT-generated T cell phenotype is able to induce proliferation of naïve T cells as well as the differentiation of Th17 cells.

## Materials and Methods

### Expression of Recombinant Protein

Recombinant PMT and PMT^C1165S^ were kindly provided by J. Orth and K. Aktories (Freiburg). The toxin was expressed and purified as described before. Possible endotoxin contaminations were removed by an endotoxin removing gel (Pierce; Hildebrand et al., 2014).

### T Cell Isolation

Peripheral blood mononuclear cells were isolated from fresh blood or buffy coats by density gradient centrifugation (Pancoll 1.077 g/mL; PAN Biotech) and washed three times with PBS. CD4-positive T cells were negatively selected (untouched) by magnetic-associated cell sorting (CD4^+^ T Cell Isolation Kit, Miltenyi Biotec) and AutoMACS technology twice. Sorted cells (93–97% purity) were cultured in RPMI 1640 medium (Biochrom AG) supplemented with 10% FBS and 1% penicillin and streptomycin (PAA laboratories) at 37°C in a humidified atmosphere in the presence of 5% CO_2_.

### Activation and Stimulation

Cells were seeded at a concentration of 2.5 million per ml and activated with anti-CD3 and anti-CD28-coated beads (T Cell Activation/Expansion Kit, Miltenyi Biotec) at a ratio of 1:2. Cells were stimulated with PMT, PMT^C1165S^ or heat-inactivated dT PMT (65°C, 1 h) at a concentration of 1 nM. Cholera toxin (CT) and nocodazole (Sigma–Aldrich) were used at 100 ng/ml.

### T Cell Differentiation *In Vitro*

For the *in vitro* differentiation of Th17 cells, CD3/CD28-activated Th cells were stimulated 5–7 days with 100 ng/ml of recombinant IL-1β, IL-6, IL-23, and TGF-β (Immuno Tools GmbH). Regulatory T cells were generated by treatment with 100 ng/ml rapamycin (Calbiochem) and 200 ng/ml IL-2 (Immuno Tools GmbH).

### Cell Viability Assay

T cells were stimulated for 5 days in a 96-well plate. Then the CellTiter-Glo^®^ Luminescent Cell Viability Assay (Promega) was performed by following the manufacturer’s protocol. By adding the CellTiter-Glo reagent, cells are lysed and a luminescent signal (measured with a microplate reader, Hidex) proportional to the amount of ATP is generated.

### Carboxyfluorescein Succinimidyl Ester (CFSE) Staining

Isolated T cells were stained with carboxyfluorescein succinimidyl ester (CFSE; 2.5 million cells/ml, 5 μM CFSE, Biolegend) for 10 min at room temperature (r.t.) in the dark, before the reaction was stopped with cold medium containing 10% FCS. After washing the cells with medium, they were activated and stimulated as described above. Fluorescence intensity of the cells was quantified with a FACSCanto on day one (basis measurement) and day five.

### ELISA

Supernatants of cultured T cells were collected at the indicated time points for the subsequent quantification of cytokines. IL-2, IL-17A, and IL-10 ELISAs (Biolegend) were performed following the manufacturer’s instructions. Measurements were performed on a Tecan GENios Pro plate reader and analyzed with Magellan5 software.

### Cell Cycle Stage Analysis

T cells were synchronized in G0 phase by culturing them overnight in the absence of an activation stimulus. Then the cells (250,000 cells per condition) were activated with anti-CD3/CD28 beads and stimulated with PMT, CT (G0/1 phase), or nocodazole (G2 phase). After 24 h cells were washed and the cell pellet was resolved in 70% ethanol and stored on ice for 15 min for fixation. Washed cells were then resolved in staining-solution (50 μg/ml propidium iodide, 100 μg/ml RNAse A, 0.02% Tween 20 in PBS) with constant vortexing. After incubation for 40 min at 37°C cells were analyzed with a FACSCanto (*x*-axis: PE-A, *y*-axis: PE-W).

### Immunoblot Analysis

Cells were washed twice and lysed as described before (Hildebrand et al., 2010) in RIPA buffer and analyzed by western blotting. Samples were boiled in Laemmli buffer and separated on a 4–20% gradient polyacrylamide gel (Anamed GmbH) by SDS-PAGE, transferred to nitrocellulose, and immunoblotted. To determine STAT activation, antibodies against p(Y705)STAT-3, p(Y693)STAT-4, STAT-4, p(Y694)STAT-5, STAT-5, p(Y641)STAT-6, STAT-6, actin, and anti-rabbit horseradish peroxidase (HRP) from Cell Signaling Technology and against STAT-3 from Santa Cruz Biotechnology were used. To determine cell cycle-relevant proteins, nuclear extracts were produced as described before (Hildebrand et al., 2010). Antibodies were purchased from Cell Signaling Technology (anti-CDK6, -CDK4, -cyclin D1, -p27, -cyclin B, p(Y15) CDK1), Santa Cruz Biotechnology (anti-cyclin E, C-19), and Sigma–Aldrich (anti-B23). Primary antibodies were used 1:1000, HRP-coupled secondary antibodies 1:5000.

### Caspase 3, 7, and 8 Activity Assay

T cells were seeded in 96-well plates, activated with anti-CD3/CD28-coated beads and treated for 1 or 5 days with PMT. Caspase 3, 7, and 8 activities were measured using the Caspase-3/7Glo and Caspase-8Glo Assay (Promega) in accordance to the manufacturer’s instructions. Luminescence was measured using the Plate CHAMELEON^TM^V microplate reader (Hidex).

### Reverse Transcriptase-PCR

RNA was extracted from T cells with the “High pure RNA Isolation Kit” (Roche), following the manufacturers protocol. Total RNA was quantified with a NanoDrop and cDNA was prepared using “Reversed Aid First Strand cDNA Synthesis Kit” (Fermentas Life Science). The cDNA was then used for quantitative PCR analysis with the “SYBR Green Rox mix” (Thermo Scientific) and sequence-specific primers: *actin*, sense, 5′- AGA GCT ACG AGC TGC CTG AC -3′, antisense, 5′- AGC ACT GTG TTG GCG TAC AG-3′, human *FOXP3*: sense 5′-GAA ACA GCA CAT TCC CAG AGT TC-3′, antisense 5′- ATG GCC CAG CGG ATG AG -3′, human *TBET*: sense, 5′- AAG TTT AAT CAG CAC CAG ACA GAG ATG ATC -3′, antisense, 5′- AAC AGA TGT GTA CAT GGA CTC AAA GTT CTC -3′, human *GATA3*: sense 5′- GCT GTC TGA AGC CAG GAG AGC -3′, antisense 5′- ATG CAT CAA ACA ACT GTG GCC AA -3′, human *RORC*: sense 5′- CCG CTG AGA GGG CTT CAC -3′, antisense 5′- TGC AGG AGT AGG CCA CAT TAC A -3′. The results were analyzed using the Fast Real-Time PCR System (Applied Biosystems). The mean values of the results (mean ± SEM; *n* = 2) were normalized to *actin*.

### FACS Staining

T cells were fixed with 4% paraformaldehyde/PBS at r.t. for 15 min. Subsequently, the cells were permeabilized in 0.1% Triton X-100/PBS for 5 min (r.t.). Afterward, one million cells were incubated with 2 μg of antibodies against CD4 (Miltenyi Biotec), RORγt, Foxp3, IL-10, or IL-17 (eBioscience) for 1 h at 4°C. After washing three times, cells were incubated for 30 min at 4°C with 1 μg of the secondary antibody per one million cells (anti-mouse FITC or anti-rat PE, eBioscience). The antibody against CD4 was directly labeled with an APC fluorophore. Washed cells were then analyzed on a FACSCanto.

### Co-culture Experiment

Isolated, CD3/CD28-activated T cells were differentiated into Th17 cells or Tregs or stimulated with PMT for 7 days. Then untouched T cells from the same donor were isolated and stained with CFSE (5 μM CFSE/DMSO for 10 min at 37°C, before the staining reaction was stopped with ice cold medium/10% FCS). CFSE-stained cells were activated with CD3/CD28-beads and co-cultured with washed Tregs, Th17 cells, or PMT-generated T cells (ratio 1:1). After 5 days of co-culture the fluorescent signal of the CFSE-labeled cells was quantified on the FACSCanto (FITC channel). Histogram overlays were performed using the Weasel.jar software.

### Statistics

Statistical significance was assessed using Prism6 software using a two-sided ANOVA multiple comparison test with ^∗^*p* ≤ 0.05, ^∗∗^*p* ≤ 0.01, ^∗∗∗^*p* ≤ 0.001, ^∗∗∗∗^*p* ≤ 0.0001.

## Results

### PMT Augments CD3/CD28-activated Th Cell Proliferation

**FIGURE 1:**
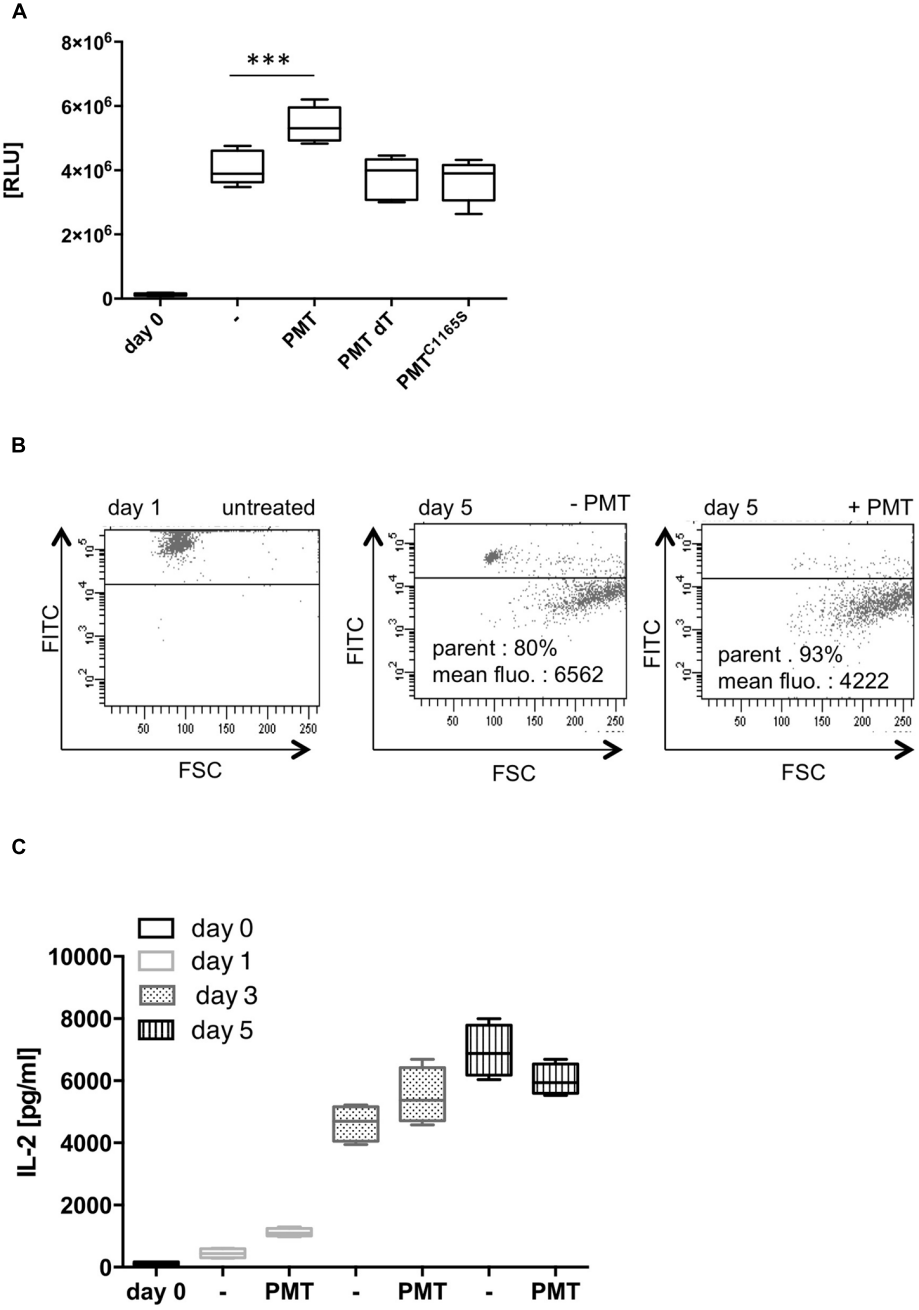
***Pasteurella multocida* toxin (PMT) stimulates CD3/CD28-activated T cell proliferation. (A)** CD4-positive T cells were activated with anti-CD3/CD28-coated beads (T Cell Activation/Expansion Kit, Miltenyi) and stimulated for 5 days with 1 nM PMT wild-type (wt), heat-inactivated PMT (dT), or a catalytically inactive mutant (PMT^C1165S^). Viability of cells was determined by quantification of ATP using the CellTiter-Glo^®^ Luminescent Cell Viability Assay (Promega). Shown is the mean ± SD, *n* = 6 (six donors, each experiment performed in duplicates). **(B)** Th cells were CFSE-labeled, CD3/CD28-activated and stimulated for 5 days with 1 nM PMT. Proliferation cycles were assayed by measuring the decreasing fluorescence by flow cytometry. The mean fluorescence and percentages refer to the gated (dividing) cells. Shown is the result of one representative donor out of three. **(C)** CD3/CD28-activated Th cells were stimulated with PMT for 1, 3, or 5 days. IL-2 ELISAs were performed (mean ± SD, *n* = 4; four donors, each experiment performed in duplicates). Statistical analysis was performed using two-sided ANOVA for multiple comparisons (^∗∗∗^*p* ≤ 0.001).

Our investigation aimed to identify whether Th cell differentiation is targeted by the bacterial toxin PMT. In the experiments, we treated blood-derived CD4-positive T cells with anti-CD3- and anti-CD28-coupled beads to achieve full activation of the cells in the absence of an APC. This activation induces a network of downstream signaling pathways that eventually leads to cell proliferation and subsequently, depending on the cytokine environment, to differentiation into specific effector cells (Coquet et al., 2015). To investigate whether PMT is toxic for T cells, we initially checked its influence on the viability of CD3/CD28-activated T cells. To test this, we quantified the amount of cellular ATP in an assay where the ATP is used to generate a luminescence signal. **Figure 1A** shows that the ATP level of CD3/CD28-activated cells was increased considerably after 7 days in culture which confirmed the activation of cells and suggested an increase of the cell number. The additional stimulation with PMT remarkably enhanced lymphocyte viability. A PMT mutant, where a point mutation within the catalytic domain renders the toxin inactive, as well as heat-inactivated PMT did not alter the CD3/CD28-mediated activation. Therefore we concluded that the observed effect is mediated by the enzymatic function of PMT and that a potential endotoxin contamination can be excluded. To investigate whether the increase in cell viability is due to an increase in Th cell proliferation, stainings with fluorescent CFSE that binds covalently to proteins and other amino groups were performed. During cell division the fluorescent dye is passed on to the dividing cells and thus the fluorescence intensity decreases with each division. **Figure 1B** shows that PMT treatment leads to an enhanced number of cells that underwent cell division. Additionally, the PMT-stimulated cells divided more frequently than the CD3/CD28-activated cells (mean fluorescence of 4222 for PMT-treated cells vs. 6562 for untreated cells).

The proliferation rate of T cells can be altered by various signals. IL-2 is a potent growth factor that mediates cell cycle entry, increases survival of dividing cells and also guides the differentiation of T cells (Oppenheim, 2007). To investigate whether PMT enhances T cell proliferation through an increased secretion of IL-2, we quantified the production of this cytokine by ELISA. The kinetics revealed that the IL-2 concentration of CD3/CD28-activated cells increased from day one to day seven, as expected. Addition of PMT effectively enhanced the release of the cytokine compared to CD3/CD28-activated cells, at least during the first 3 days of culture, which might be a reason for the observed increase in proliferation (**Figure 1C**).

### PMT Mediates Cell Cycle Progression and Survival of Th Cells

**FIGURE 2:**
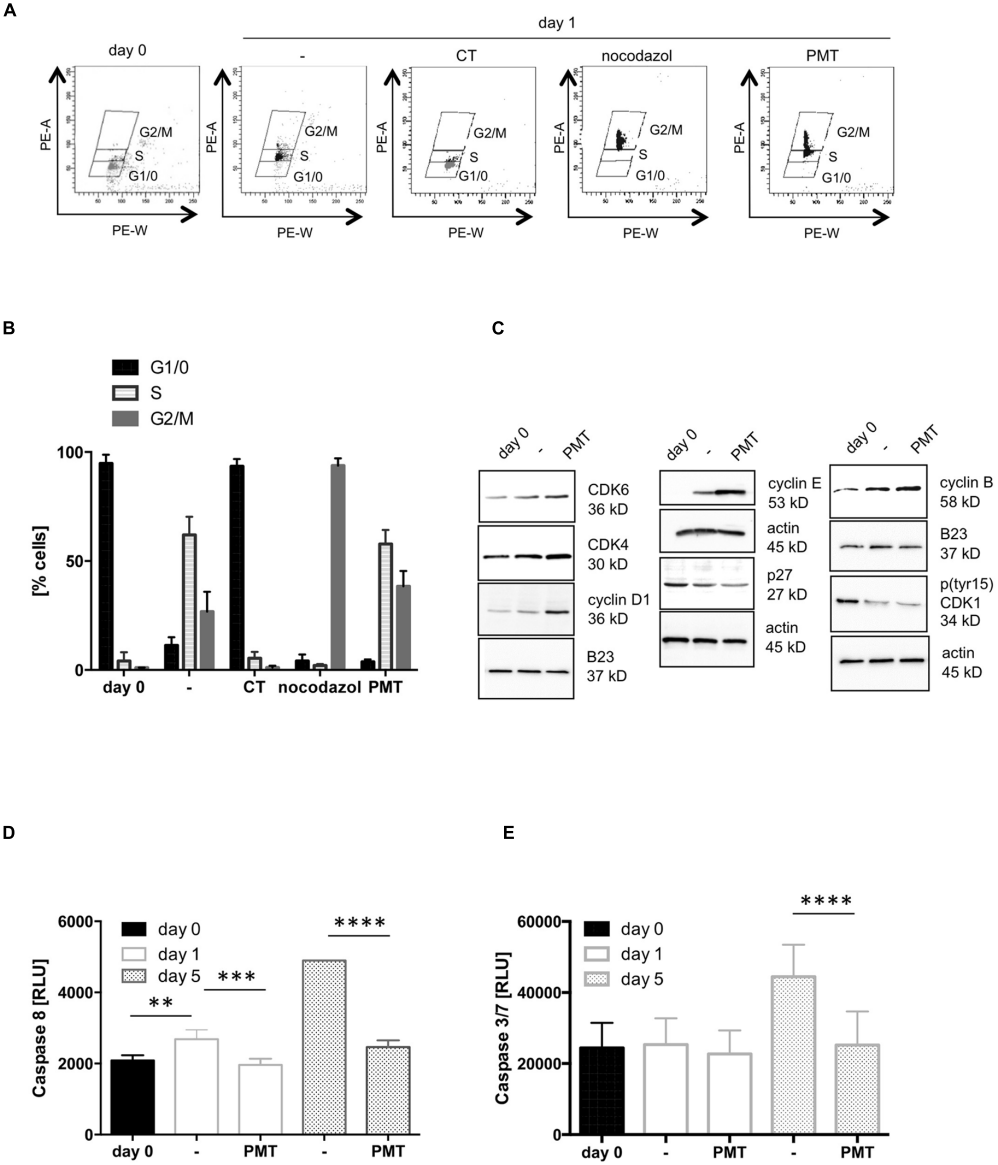
**PMT stimulates cell cycle progression. (A)** CD4-positive T cells were cultured overnight without activation to synchronize cells in G0 phase. Then cells were CD3/CD28-activated and stimulated with PMT for 24 h. As controls, cells were treated with 100 ng/ml CT (G0/1 phase) or 100 ng/ml nocodazole (G2 phase). Nuclei were stained with propidium iodide (PI) and intensity of the PI signal was quantified by flow cytometry (*x*-axis: PE-A, *y*-axis: PE-W). **(B)** Quantification of the percentage of cells in G1/0, S and G2/M phase (mean ± SD, *n* = 3). **(C)** Cell lysates were used for western blot analysis with specific antibodies against cyclin B, pCDC2 (CDK1), cyclin D1, cyclin E, CDK4, or p27. Equal loading was verified by detection of actin (whole cell lysates) or B23 (nuclear extracts). The results were corroborated with two more donors. **(D,E)** T cells were stimulated with PMT for 1, 3, or 5 days, lysed and the activation of caspase-8 **(D)** or caspases 3 and 7 **(E)** was measured with a Caspase-8Glo^®^ Assay or a Caspase 3/7-Glo^®^ Assay, respectively (Promega). Shown is the mean ± SD, *n* = 3 donors. Statistical analysis for this figure **(B,D,E)** was performed using two-sided ANOVA for multiple comparisons (^∗∗^*p* ≤ 0.01; ^∗∗∗^*p* ≤ 0.001; ^∗∗∗∗^*p* ≤ 0.0001).

In the following experiments we aimed to determine the influence of PMT on cellular proliferation in more detail. Therefore we analyzed the cell cycle stages of Th cells. The cell cycle can be divided into four major phases. In the G0 phase the cell rests. When it enters G1 phase it increases its size and produces proteins as well as other cellular components. DNA replication takes place in the synthesis (S) phase, which is followed by the preparation (G2 phase) for cell division. Finally the division of the nucleus (M Phase, mitosis) and the formation of two daughter cells (cytokinesis) occur. An effective T cell cycle run requires TCR activation to trigger transition from the G0 to G1 phase and subsequently activation of the IL-2 receptor, which mediates the entry into S phase (Cantrell and Smith, 1984; Meuer et al., 1984). To examine the effect of the toxin on the cell cycle, we quantified the DNA content of the cells. Cells were stained with propidium iodide (PI) to determine the amount of cellular DNA by FACS. As a control for G2/M, cells were treated with nocodazole, a substance that interferes with the polymerization of microtubules and thus inhibits division into daughter cells so that nocodazole-treated cells contain the maximum amount of DNA during cell cycle. As a control for G0/1 phase and therefore the minimal amount of DNA, cells were treated with CT from *Vibrio cholerae* which is known to induce cell cycle arrest in the early G1 phase (Zheng et al., 2014). **Figures 2A,B** show that non-activated Th cells paused in G1/0 phase. CD3/CD28-activation stimulated the increase of DNA per cell which is characteristic for the S phase of the cycle. Stimulation of CD3/CD28-activated cells with PMT induced a further increase of the PI signal and revealed a population with a DNA amount that is typical for the G2/M phase. We therefore conclude that the toxin mediates progression of the cell cycle and DNA synthesis. To link this to molecules known to be involved in cell cycle progression, we checked the impact of PMT on the expression and activation level of cyclin-dependent kinases (CDKs), the regulatory cyclins and CDK inhibitors (**Figure 2C**). CDK activity is regulated by its phosphorylation status, association with cyclins and binding of inhibitory molecules. Entry into S phase is mediated by CDK6/cyclin D and CDK4/cyclin D complexes and their activation requires the degradation of the inhibitor p27. The active complex then enables transcription factors to activate G1/S-phase gene expression (Bendris et al., 2015). The performed western blot analysis showed that PMT indeed mediates an increased degradation of p27, an increase in cyclin E expression and additionally induces a stronger accumulation of CDK4, CDK6, and cyclin D in the nucleus than CD3/CD28. Furthermore PMT increases the expression of cyclin E which in complex with CDK2 promotes degradation of p27 (Bendris et al., 2015). Hence, toxin treatment seems to promote the entry into the S phase of the cell cycle. The entry of all eukaryotic cells into mitosis is regulated by activation of CDK1 (cdc2) at the G2/M transition. A critical regulatory step is the dephosphorylation of CDK1 at Thr14 and Tyr15 (Atherton-Fessler et al., 1994) and the phosphorylation-regulated translocation of cyclin B1 to the nucleus. Our results show reproducibly that PMT slightly enhances the translocation of cyclin B to the nucleus and a dephosphorylation of Tyr15 of CDK1. These results corroborate earlier findings reporting the upregulation of cell cycle progression by PMT using a fibroblast cell line (Wilson et al., 2000).

From our previous work in HEK 293 cells we knew that PMT is not only able to trigger proliferation, but also protects cells from staurosporine-induced apoptosis through the PI3K-dependent phosphorylation of Akt and the constitutive expression of the survival kinase Pim-1, respectively (Preuss et al., 2010). Programmed cell death is an integral part of the immune response, as the removal of T cells after clearance of the infection restores immune cell homeostasis (Pinkoski and Green, 2005). Taking this into consideration, the increased T cell number under PMT treatment could additionally be caused by the inhibition of apoptosis of activated T cells. To check this possibility, we analyzed caspase-8 and caspase-3/7 activation as central markers of the apoptotic response. The luminescence-based assays show that the activity of caspase-8 (**Figure 2D**) and caspase-3 and -7 (**Figure 2E**) increased considerably after 5 days of CD3/CD28-activation. Stimulation with PMT, however, kept the activity of the death caspases at the basal level of freshly isolated, naïve T cells. This supports our hypothesis that the toxin does not only trigger proliferation but also inhibits apoptosis of receptor-activated Th cells. In summary these data demonstrate that PMT expands CD3/CD28-activated Th cells by triggering proliferation and by suppressing apoptosis.

### PMT Induces Foxp3 and RORγt-Positive T Cells

**FIGURE 3:**
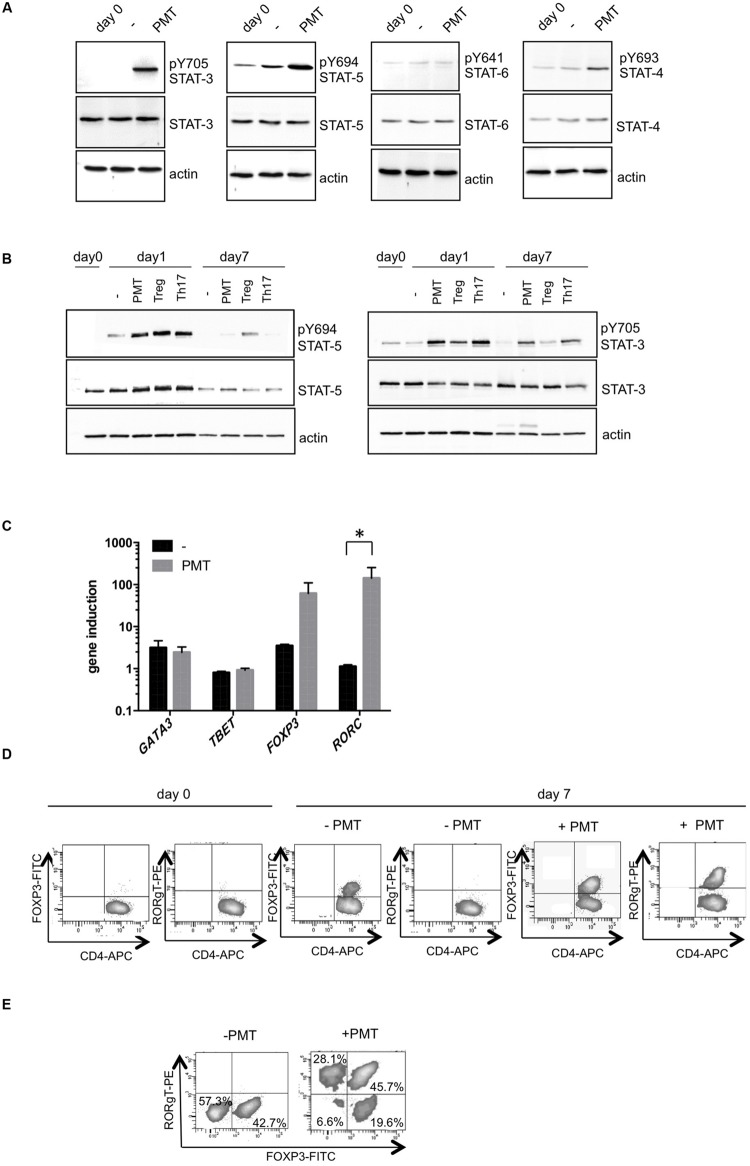
**PMT induces Foxp3 and RORγt-positive T cells.** CD3/CD28-activated T cells were stimulated with PMT overnight or for 7 days, respectively. **(A)** Whole cell lysates were produced and antibodies against p(Y705)STAT-3, STAT-3, p(Y694)STAT-5, STAT-5, p(Y641)STAT-6, STAT-6, and p(Y693)STAT-4, STAT-4 were used for immunoblotting. Equal loading of protein was verified by actin detection. **(B)** As a positive control, T cells were treated with 100 ng/ml IL-1β, IL-23, IL-6, TGF-β (Th17 cells), or 100 ng/ml IL-2 and rapamycin (Tregs). **(C)** Quantitative RT-PCRs were performed using SYBR Green and sequence specific primers for *FOXP3, TBET, GATA3*, and *RORC*. Results were normalized against the housekeeping gene *actin*. Shown is the mean of induction compared to non-activated cells. SD, *n* = 2 donors, experiment performed in duplicates. Statistical analysis was performed using two-sided ANOVA for multiple comparisons (^∗^*p* ≤ 0.05). **(D)** Intracellular FACS analysis. Fixed and permeabilized cells were stained with anti-CD4-APC, purified anti-Foxp3 or anti-RORγt and FITC-/PE-labeled secondary antibodies. **(E)** Intracellular double stain of Foxp3 or RORγt of cells on day ten. Data shown in **(A,C,D)** are representative for three, and **(E)** for two experiments.

As we had observed that PMT supports the CD3/CD28-mediated proliferation of Th cells, we next aimed to investigate whether the toxin also modulates the differentiation of naïve T cells into specific T cell subtypes. T cell differentiation is dependent on the cytokine environment. Downstream of cytokine-induced receptor activation, STAT transcription factors play a pivotal role in T cell differentiation through the induction of subtype-specific genes. Hence our first question addressed the PMT-mediated phosphorylation and activation of STAT family members. Western blot analysis of toxin-stimulated cells (**Figure 3A**) shows a pronounced phosphorylation of STAT-3 in comparison to CD3/CD28-induced cells after 24 h of toxin treatment. As expected, STAT-5 phosphorylation was induced by CD3/CD28 activation, presumably via the production of IL-2, but this activation level could still be enhanced by PMT treatment. Phosphorylation of STAT-4 was not affected by antibody-activation and only slightly induced by PMT. Finally STAT-6 phosphorylation could not be observed under antibody or toxin conditions. As pSTAT-3 mediates differentiation of Th17 cells and pSTAT-5 is important in Treg formation, we generated these T cell subtypes *in vitro* and compared the STAT activation level of cytokine-differentiated control cells with PMT-treated cells. On day one, PMT treatment caused an intense phosphorylation of both transcription factors, however, on day seven only pSTAT-3 was detectable. Whereas fully differentiated (day 7) Tregs contained activated STAT-5 and only basal level of phosphorylated STAT-3, Th17 cells showed high STAT-3 but no STAT-5 activation. The data show that PMT-stimulated T cells display the same STAT activation pattern like Th17 cells (**Figure 3B**). When we analyzed the master transcription factors of subtype development after 24 h of treatment (**Figure 3C**), we found that in line with our previous results, toxin-stimulated CD4-positive cells contained higher copy numbers of *RORC* (STAT-3 target) and *FOXP3* (STAT-5 target) mRNA compared to *GATA3* (induced by STAT-6) and *TBET* (induced by STAT-4). This was confirmed on the protein level by intracellular FACS analysis (**Figure 3D**) where high expression of Foxp3 and RORγt could be found in cells that had been treated with PMT for 7 days. The CD3/CD28-activated population already contained a considerable amount of Foxp3, which is probably due to IL-2-mediated STAT-5 activation. These data raised the question whether PMT stimulates the differentiation of two different T cell subtypes or whether one subtype with two simultaneously expressed master transcription factors was produced. Double stainings for Foxp3 and RORγt (**Figure 3E**) revealed that the antibody-activated T cell population did not contain any double positive cells. In contrast to that the PMT-treated population contained around 30% RORγt-positive, 20% Foxp3-positive and almost 46% double positive T cells, even after 10 days of culture.

### PMT Mediates Differentiation of an Inflammatory T Cell Subtype

**FIGURE 4:**
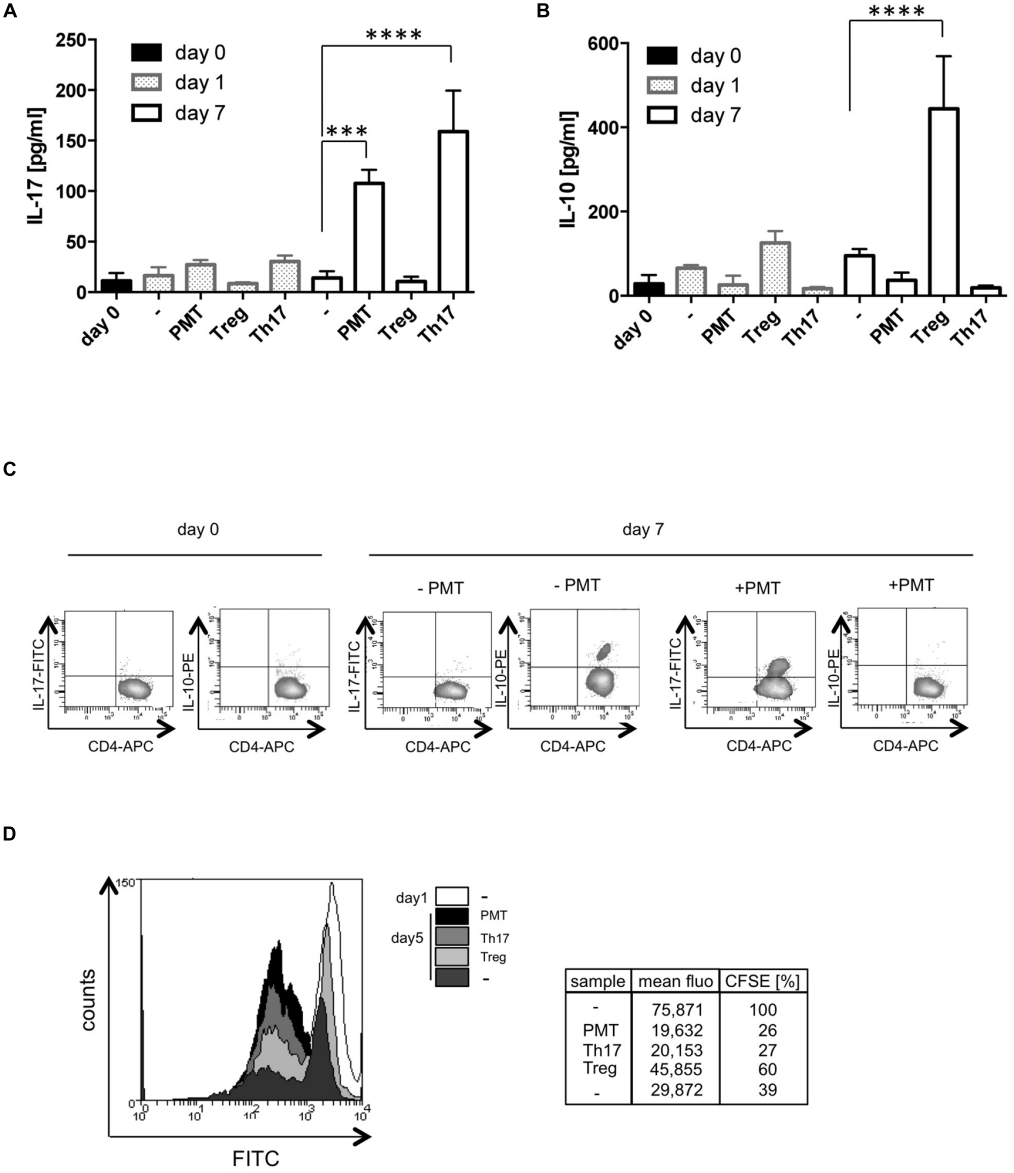
**PMT mediates a Th17 phenotype.** CD3/CD28-activated Th cells were stimulated with PMT for the indicated time points. In **(A)** IL-17A and **(B)** IL-10 ELISAs were performed (mean ± SD, *n* = 3). Statistical significance of the produced cytokine was compared to the value obtained on day 0 using two-sided ANOVA for multiple comparisons (^∗∗∗^*p* ≤ 0.001; ^∗∗∗∗^*p* ≤ 0.0001). **(C)** For intracellular FACS analysis, fixed and permeabilized cells were double-stained with anti-CD4-APC and purified anti-IL-10 or anti-IL-17 antibodies and the according FITC-/PE-labeled secondary antibodies. **(D)** Th cells were stimulated with PMT for 7 days. These PMT-generated T cells were then co-cultured with freshly isolated, CFSE-labeled Th cells (ratio 1:1) from the same donor. The fluorescent signal of the FITC-positive cells was quantified on day one and on day five of co-culture. Data shown in **(C,D)** are representative for three experiments (three donors).

Our results pointed to two different sets of T cell responses toward the bacterial toxin. Th17 cells have an autoinflammatory potential and support the immune response against extracellular bacteria, whereas Foxp3-positive Tregs have a suppressive role and protect the organism from autoimmunity. The different effector T cell types are ultimately defined by the cytokines that they release and by their function. Therefore we investigated which cytokines are actually released from PMT-stimulated cells and whether they support or suppress a T cell-driven immune response. ELISA analysis revealed that PMT-modulated cells release high amounts of IL-17A that is almost comparable to that of Th17 cells (**Figure 4A**). However, these cells produced nearly no immunosuppressive IL-10 (**Figure 4B**). FACS data corroborated these findings and additionally showed that there is no defect in the release of cytokines, but that IL-10 is hardly expressed by these lymphocytes (**Figure 4C**). Some of the CD3/CD28-activated T cells produced IL-10 (**Figures 4B,C**), which confirmed the differentiation of a few functional Foxp3-positive cells. To investigate whether the PMT-generated T cell subtype would rather have an activated or suppressive phenotype, we measured the ability of PMT-modulated T cells to increase or inhibit proliferation of CD3/CD28-activated CFSE-labeled CD4-positive T cells. Our data show that the CFSE signal of CD4-positive responder cells co-cultured with the PMT-differentiated effector subtype was lower than the signal obtained by CD3/CD28-activated responders. Additionally, the influence of PMT effector cells on proliferation was similar to the impact of Th17 cells. In contrast to this, Tregs showed an inhibitory impact on T cell proliferation (**Figure 4D**). This experiment suggests that the toxin modulates lineage decision of CD3/CD28-activated T helper cells toward an inflammatory subtype.

## Discussion

The relationship between the host and microbial pathogens can be quite complex. On the one hand the higher organism uses a highly specialized immune system for the detection and control of invading microorganisms, whereas pathogens have developed various strategies to avoid their recognition and elimination. The host’s innate immune system can sense foreign structures through PRRs. In consequence of this recognition, pathogen-induced phagocytosis and presentation on MHC takes place. Presentation of the antigens to the TCR and the resulting association of CD3 with the TCR-peptide-MHC complex transmits the activation signal to intracellular molecules, which trigger a cascade of biochemical events that result in the expansion of antigen-specific T lymphocytes. In addition, the differentiation into effector types is crucial for the course of the immune response. Depending on the microbe and the activated PRRs, a specific set of cytokines is produced by the APC, which mediates the differentiation of appropriate effector types. The plasticity of Th cell differentiation during infection allows the development of pathogen-specific effector T cells. However, also the invading microorganism may profit from this plasticity by directing the differentiation to an ineffective or a suppressive effector type. One example for such a manipulation has been described for *Chlamydia muridarum*. Chlamydia-infected APCs are modulated in terms of the phenotype, cytokine secretion profile and antigen presentation. This eventually shifts the immune response from a protective Th1 to a non-protective Th2 response (Kaiko et al., 2008). Also extracellular bacteria such as *Staphylococcus aureus* and *Streptococcus pyogenes* disturb the balance between activation and inhibition of immune cells that protects the host from an over-reaction and re-establishes homeostasis after clearing the infection. Superantigens (SAgs) produced by these bacteria can activate up to 20% of all T cells in an individual, mediate a vast T cell expansion and induce a strong cytokine storm that causes serious harm to the host. Eventually, the majority of activated T cells die and the remaining cells will be anergic or even induce SAg-specific Treg-mediated tolerance to the microbe (Papiernik, 2001).

An excellent target to modulate the differentiation toward an ineffective T cell subtype are STAT transcription factors that transmit the cytokine-encoded differentiation signal. PMT activates the JAK-STAT pathway very efficiently in many types of cells (Kubatzky et al., 2013) and we found that the toxin also induces STAT activity in human T cells. After 1 day of PMT stimulation, the phosphorylation of both, STAT-3 and STAT-5 was very pronounced, whereas after 7 days of stimulation only phosphorylation of STAT-3 was detectable. Compatible with these results, RT-PCRs revealed a high expression of STAT-5-dependent *FOXP3* and STAT-3-promoted *RORC* in PMT-treated cells. This suggested that differentiation of Tregs and Th17 cells had taken place. Although the parallel development of two opposing subtypes appears paradox at first glance, it has been hypothesized that the interplay between iTregs and Th17 cells actually fine-tunes the activation to prevent hyperactivation and to facilitate the return to the steady state. This is supported by the fact that the development of these two subtypes is closely connected. Both subtypes need TGF-β for differentiation and the final direction of differentiation is induced by additional cytokines. TGF-β, in cooperation with IL-2-signaling, promotes STAT-5 activation and induction and maintenance of *FOXP3* via the induction of the Smad pathway. Nevertheless, TGF-β also mediates the maintenance of a functional IL-6 receptor on activated T cells. Under inflammatory conditions, with high concentrations of IL-6, TGF-β enables IL-6/STAT-3 signaling and eventually the differentiation of Th17 cells (Lee et al., 2009). The host’s benefit from a high plasticity of Treg and Th17 cells is probably a fast reaction to the course of an infection. After the initial recognition of the pathogen, the inflammatory conditions change from the tolerogenic phenotype of nTregs toward the response-activating Th17 cells. In the process of clearance the cells are able to transdifferentiate again into iTregs to protect the body from hyperactivation (Gagliani et al., 2015). In consideration of this close connection of Th17 cells and Tregs and the complexity of the cytokine environment during infection it is not surprising that naïve, TGF-β-activated T cells can co-express RORγt and Foxp3. Our experiments show that after 1 week of culture several CD3/CD28-activated T cells are Foxp3 positive. This is probably due to the CD28-triggered and IL-2 mediated promotion of Foxp3 expression (Burchill et al., 2007; Guo et al., 2008) and may be due to an increased survival of nTregs (Weiss et al., 2011), (**Figure 3D**). Interestingly, 45% of the PMT-stimulated activated T lymphocytes are positive for both transcription factors. This could be caused by the strong activation of STAT-3 and STAT-5 during the first hours of stimulation. Furthermore it suggests that the observed toxin-mediated protection from apoptosis expands primarily Foxp3-positive cells, which later additionally express RORγt. The parallel production of RORγt and Foxp3 can lead to the induction of both IL-10 and IL-17 or just one of them (McGeachy et al., 2007; Ichiyama et al., 2008; Zhou et al., 2008) and Foxp3 can directly bind to RORγt and prevent its action. Only the additional stimulation with inflammatory cytokines such as IL-6 relieves Foxp3-mediated inhibition through a STAT-3-dependent suppression of *FOXP3* expression (Yao et al., 2007), and promotes differentiation of Th17 effector cells (Ichiyama et al., 2008; Zhou et al., 2008). Our experiments indicate that the release of IL-17 increases from day one to day seven of PMT stimulation whereas the IL-10 concentration did not. This suggests that in the beginning of PMT-stimulation the activation of STAT-3 and STAT-5 mediates the development of some IL-17 releasing Th17-cells, some IL-10 releasing Tregs, and some RORγt, Foxp3 double positive cells, which probably do not produce IL-17 due to an inhibitory interaction. At later time points, when only STAT-3 but no STAT-5 activation is observable, RORγt is released from Foxp3 inhibition and effectively induces IL-17 expression. An additional explanation for the low IL-10 production of toxin-treated cells could be the expression of the kinase Pim-1. While the expression of Pim-1 is inhibited by TCR activation, inflammatory conditions with high level of IL-6 lead to the induction of Pim-1 in human Tregs (Li et al., 2014). The kinase then phosphorylates Foxp3 at serine 422, which inhibits Foxp3 chromatin binding activity and eventually inhibits IL-10 production. Interestingly, our studies in HEK 293 cells show that PMT is a much more potent inducer of Pim-1 than IL-6 (Hildebrand et al., 2010). Therefore PMT stimulation could overcome the TCR-mediated inhibition of Pim-1 in the Foxp3-positive cells and the following mediated phosphorylation would suppress IL-10 production.

An interesting connection between Th17 cells and the bacterium is the interaction between the bone system and the immune system. IL-17 is involved in the excessive inflammation and activation of osteoclasts that strongly contributes to bone destruction associated with rheumatoid arthritis (Lubberts, 2015). Here, Th17 cells trigger the expression of receptor activator of nuclear factor-κB ligand (RANKL) in synovial cells, which then, in cooperation with other inflammatory cytokines, stimulate the differentiation and activation of monocytes into osteoclasts (Sato et al., 2006). PMT is known to be a mitogen for osteoclasts (Martineau-Doize et al., 1993; Jutras and Martineau-Doize, 1996) and pigs infected with *P. multocida* suffer from atrophic rhinitis, a pathologic condition characterized by PMT-stimulated osteoclastic bone resorption at the nasal turbinates and an inflammation of the nasal mucosa (Wilkie et al., 2012). We have previously shown that the PMT-triggered differentiation of bone marrow-derived mouse macrophages into osteoclasts is strongly dependent on cytokines released by B cells (Hildebrand et al., 2011). Cytokines produced by Th17-cells could additionally support the PMT-mediated osteoclast formation and the destruction of bone and thereby support the pathogenesis of toxigenic *P. multocida* strains. Nevertheless, the direction toward a Th17-mediated response is the normal course of an immune response against pulmonary and gastrointestinal pathogens like *P. multocida* (Garrido-Mesa et al., 2013). However, it has been suggested that pathogens that trigger a persistent Th17 response because they have not been not sufficiently cleared through the host’s immune system, may be more beneficial for the microorganism than the host (van de Veerdonk et al., 2009).

In summary, we show that the bacterial toxin PMT enhances the proliferation and survival of CD3/CD28-activated Th cells and that the toxin manipulates the differentiation toward a Th17-subtype through the activation of STAT transcription factors. This once more illustrates how bacteria are able to target the high plasticity of T cell subtypes to reinforce their pathogenicity and possibly to gain an advantage in terms of survival and reproduction.

## Author Contributions

KK, KH, and DH designed the study. DH and KK wrote the final manuscript. DH performed the experiments. All authors read the manuscript and discussed the results.

## Conflict of Interest Statement

The authors declare that the research was conducted in the absence of any commercial or financial relationships that could be construed as a potential conflict of interest.
